# A Comprehensive Physiological, Molecular, and Multi‐Omics Analysis Reveal Distinct Cold Stress Response Strategies in *Glycine max* Cultivars

**DOI:** 10.1111/pce.70132

**Published:** 2025-08-18

**Authors:** Dinh Nhan Lai, Yun Zhou

**Affiliations:** ^1^ Department of Botany and Plant Pathology Purdue University West Lafayette Indiana USA; ^2^ Purdue Center for Plant Biology Purdue University West Lafayette Indiana USA

Cold stress presents a major limitation to plant growth and development, significantly reducing crop productivity. Low temperatures can physically damage plant tissues and disrupt essential physiological and biochemical processes across both vegetative and reproductive organs (Yadav 2010). For example, cold stress impairs phosphorylation and respiration in leaves, inhibits nutrient and mineral uptake in roots, and induces oxidative stress, lipid peroxidation, and loss of cellular water retention, ultimately leading to necrosis or cell death (Thakur et al. 2010; Manasa et al. 2022; Gusain et al.^2023^; Ding et al. 2024). To survive such conditions, plants have evolved adaptive responses, including the scavenging of reactive oxygen species (ROS) and the accumulation of osmoprotectants such as proline and soluble sugars (Manasa et al. 2022). At the molecular level, cold stress activates signaling cascades involving key transcriptional regulators, such as C‐repeat binding factors (CBFs), which mediate the expression of cold‐responsive (COR) genes (Miura and Furumoto 2013). Upstream of the CBFs, the ICE1 transcription factor plays a central role in regulating their expression and integrating signals from broader hormonal pathways (Chinnusamy et al. 2003; Kim et al. 2015). Although much of this regulatory framework has been elucidated in the model plant *Arabidopsis thaliana*, gaining species‐specific insights is essential for improving cold tolerance and enhancing yield and biomass production in target crop species.

Soybean (*Glycine max*) is a globally important crop, valued for its high protein and oil content. As its cultivation expands into regions with diverse latitudes, soybean is increasingly exposed to cold stress, particularly during early developmental stages, posing a threat to growth and yield (Alsajri et al. 2019). Despite its agronomic significance, the molecular mechanisms underlying cold response, adaptation, and tolerance in soybean remain incompletely understood. In a recent study, Bukhari et al. (2025) systematically reveal distinct cold response strategies between two soybean cultivars, Huaxia3 (HX3, adapted to low latitudes) and Heihe43 (HH43, adapted to high latitudes), through a combination of morphological and physiological phenotyping and comparative transcriptomic and proteomic analyses across multiple time points. The study highlighted the biochemical, molecular, and cellular signatures underlying the greater tolerance of HX3 to short periods of chilling stress compared to HH43 (Figure 1). This study also identified candidate genes and regulatory modules for future functional studies and potential application in marker‐assisted breeding for cold tolerance in soybean (Figure 1).

**Figure 1:**
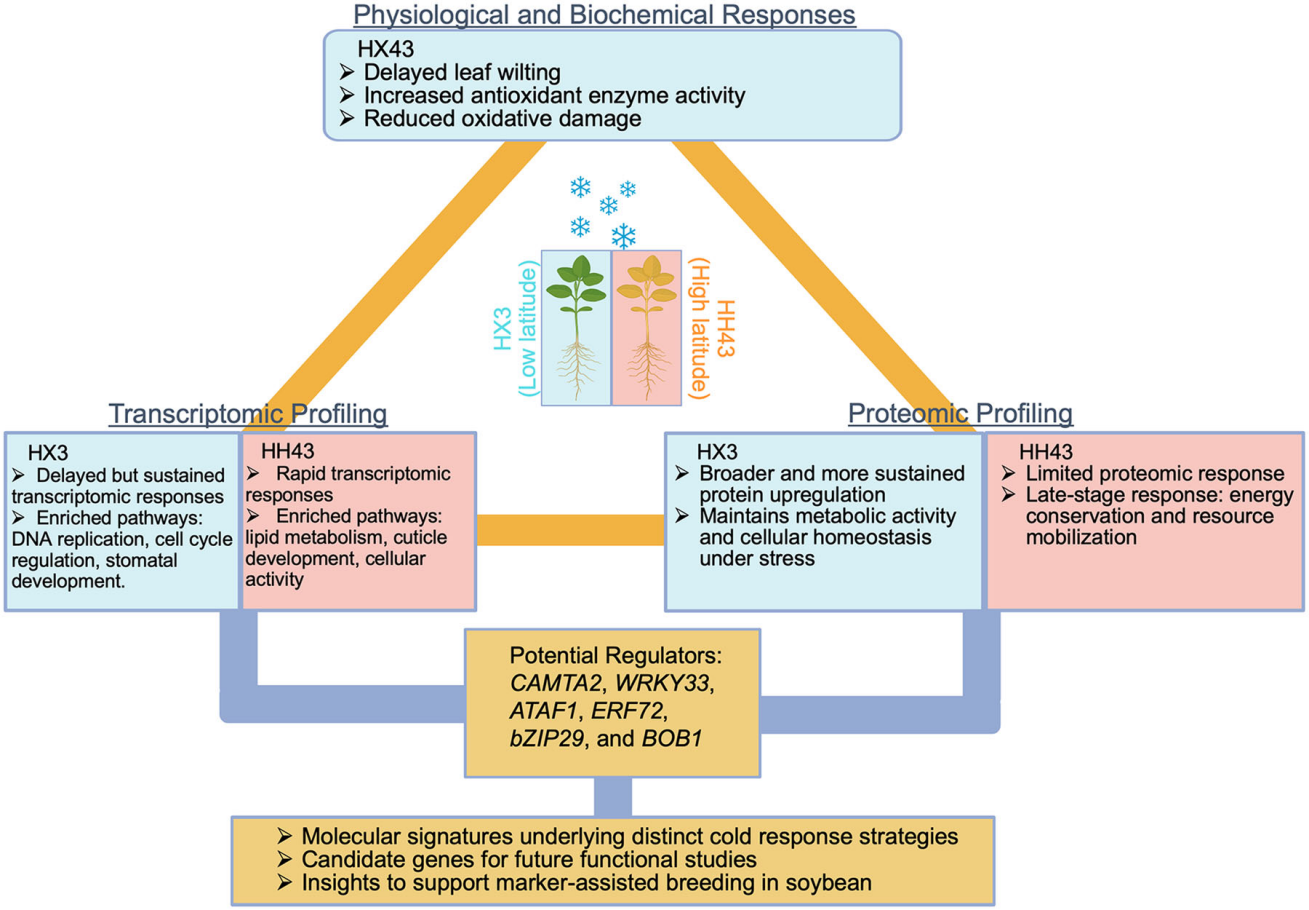
Diagram summarizing the experimental approaches and key findings of the study by Bukhari et al. 2025. Red: HH43; cyan: HX3. The figure was created using BioRender and modified in PowerPoint for clarity.

Interestingly, physiological and biochemical characterizations revealed that, despite originating from the warmer low‐latitude region, HX3 exhibited higher cold tolerance than HH43, which originates from the colder high‐latitude region (Bukhari et al. 2025). This interesting observation likely reflects long‐term adaptations of HX3 to its native environments, which are characterized by greater day‐to‐day temperature fluctuations (Duan et al. 2012; Xu et al. 2020). Following just 1 h of exposure to chilling stress (4°C), HH43 displayed leaf wilting, whereas HX3 maintained normal leaf morphology for up to 6 h under the same conditions. Moreover, HH43 experienced more severe lipid peroxidation and oxidative stress, impairing cell viability, as indicated by higher levels of malondialdehyde (MDA) and ROS at 6 h. In contrast, HX3 accumulated higher levels of osmoprotectants, such as proline and soluble sugars, and produced greater amounts of ROS‐scavenging enzymes such as SOD and CAT (Bukhari et al. 2025). Principal component and correlation analyses of physiological and biochemical traits further supported these findings: HH43 clustered closely with markers of oxidative damage (e.g., MDA), while HX3 was more closely associated with stress‐protective traits, including proline, SOD, CAT, and soluble sugars. Overall, HH43 exhibited greater sensitivity to chilling stress, whereas HX3 showed stronger adaptive responses that contribute to its enhanced cold tolerance.

Transcriptomic profiling across multiple time points following cold exposure further revealed molecular differences in cold response and tolerance between HH43 and HX3 (Bukhari et al. 2025). Analysis of leaf transcriptomes showed that, in addition to a shared set of differentially expressed genes (DEGs), each cultivar exhibited distinct transcriptional responses to chilling stress. HH43 exhibited a rapid transcriptional response as early as 1 h after treatment, whereas HX3 displayed a progressive and more sustained transcriptional reprogramming (Figure 1). Under prolonged cold stress, HX3 showed a gradual increase in DEG numbers and a greater overall DEG count at later time points (24 and 48 h) (Bukhari et al. 2025). These findings are consistent with previous studies that delayed but sustained transcriptional activation supports continued physiological adjustments and ultimately confers greater stress tolerance (Tyczewska et al. 2016; Dong et al. 2021). Thus, the extended transcriptomic response of HX3 likely contributes to its enhanced cold tolerance.

Furthermore, gene ontology (GO) enrichment analysis and Kyoto Encyclopedia of Genes and Genomes (KEGG) pathway enrichment analysis revealed distinct transcriptional responses to chilling stress in the two cultivars (Bukhari et al. 2025). HH43 showed early protective responses centered on lipid metabolism and cuticle development, followed by adjustments in cellular activities at later stages of stress. In contrast, HX3 exhibited a delayed but more comprehensive response, involving pathways related to DNA replication, cell cycle regulation, stomatal development, and various metabolic processes, including cyanoamino acid, phenylpropanoid, and carbohydrate metabolism (Figure 1). The authors also performed weighted gene coexpression network (WGCN) analysis, which identified two distinct gene modules (MEblue and MEgreen) as positively correlated with chilling stress. In parallel, gene regulatory network analysis using the GENIE3 algorithm elucidated key transcription factor (TF) networks involved in the cold stress response, including soybean homologs of *CAMTA2*, *WRKY33*, *ATAF1*, *ERF72*, and *bZIP29* (Figure 1). Future functional characterization of these TFs will enhance our understanding of the molecular networks governing cold stress responses and how plants coordinate gene expression to confer cold tolerance in crops (Figure 1).

To gain a comprehensive understanding of molecular responses to cold stress, the authors further performed proteomic profiling of the two cultivars at different time points and compared the resulting proteomic data with transcriptomic datasets (Bukhari et al. 2025). As expected, only a partial overlap was observed between differentially expressed proteins (DEPs) and DEGs across time points in both cultivars, highlighting the complexity of posttranscriptional regulation during cold stress conditions. Analysis of protein abundance profiles revealed significant differences between HX3 and HH43 during chilling stress. KEGG pathway analysis and GO analysis of DEPs further indicated different response strategies between the cultivars. Overall, HX3 exhibited broader and more sustained upregulation of proteins involved in maintaining metabolic activity and cellular homeostasis, likely contributing to its enhanced ability to cope with cold stress. In contrast, HH43 showed a more limited proteomic response, suggesting a strategy focused on energy conservation and resource recycling. Interestingly, a homolog of BOB1, which regulates organ growth and development in *Arabidopsis*, was the only protein consistently upregulated during the early response in HH43 and the sustained response in HX3, making it a promising candidate for future studies (Figure 1).

In conclusion, this study advances our understanding of cold tolerance strategies in soybean by comparing two cultivars with distinct physiological and molecular responses to chilling stress. Comparative transcriptomic and proteomic analyses identified key pathways and regulatory factors, particularly transcription factors, involved in the cold response (Figure 1). Future investigations into the roles and spatiotemporal regulation of these molecular components will further elucidate the advantages conferred by sustained transcriptional reprogramming. Moreover, integrating existing transcriptomic and proteomic datasets with analyses of genome variation among soybean cultivars may uncover the molecular basis of distinct gene regulatory networks underlying cold stress responses. Taken together, this study provides valuable insights and lays a strong foundation for developing cold‐tolerant soybean cultivars.

## Conflicts of Interest

The authors declare no conflicts of interest.

## Data Availability

This commentary does not present any new data. All discussions are based on the published datasets and analyses reported in Bukhari et al. Plant Cell & Environment 2025.
